# Inflammasome: structure, biological functions, and therapeutic targets

**DOI:** 10.1002/mco2.391

**Published:** 2023-10-09

**Authors:** Yali Dai, Jing Zhou, Chunmeng Shi

**Affiliations:** ^1^ Institute of Rocket Force Medicine State Key Laboratory of Trauma and Chemical Poisoning Army Medical University Chongqing China; ^2^ Institute of Immunology Army Medical University Chongqing China

**Keywords:** AIM2, function, inflammasome, NLRC4, NLRP3, structure, therapy

## Abstract

Inflammasomes are a group of protein complex located in cytoplasm and assemble in response to a wide variety of pathogen‐associated molecule patterns, damage‐associated molecule patterns, and cellular stress. Generally, the activation of inflammasomes will lead to maturation of proinflammatory cytokines and pyroptotic cell death, both associated with inflammatory cascade amplification. A sensor protein, an adaptor, and a procaspase protein interact through their functional domains and compose one subunit of inflammasome complex. Under physiological conditions, inflammasome functions against pathogen infection and endogenous dangers including mtROS, mtDNA, and so on, while dysregulation of its activation can lead to unwanted results. In recent years, advances have been made to clarify the mechanisms of inflammasome activation, the structural details of them and their functions (negative/positive) in multiple disease models in both animal models and human. The wide range of the stimuli makes the function of inflammasome diverse and complex. Here, we review the structure, biological functions, and therapeutic targets of inflammasomes, while highlight NLRP3, NLRC4, and AIM2 inflammasomes, which are the most well studied. In conclusion, this review focuses on the activation process, biological functions, and structure of the most well‐studied inflammasomes, summarizing and predicting approaches for disease treatment and prevention with inflammasome as a target. We aim to provide fresh insight into new solutions to the challenges in this field.

## Graphical abstract

Here, we reviewed the activation process and basic structure of inflammasomes in both inactive and active states. Biological functions of inflammasomes in this review focus on the release of proinflammatory cytokines and the activation of gasdermins, as well as its participation in multiple diseases. The complexity of its activation, structure, and functions provide numerous targets for therapy in these diseases.

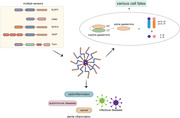

## 1 INTRODUCTION

Inflammasome is a protein complex that plays an important role in innate immunity and body's response to attacks from both pathogens and endogenous dangers signals. Inflammasome as a whole is composed of three functional components, the sensor, the adaptor and caspases downstream. The known pattern recognition receptors (PRRs) in innate immunity, the first line of defense in human immunity, include Toll like receptors (TLRs), RIG‐I like receptors (RLRs), AIM2 like receptors (ALRs), NOD like receptors (NLRs), and cyclic GMP‐AMP synthase (cGAS)/STING. NLRs family, which contains NLRP1, NLRP2, NLRP3, NLRP6, NLRC4, and NLRP12, can act as the sensor of inflammasome complex. ALRs family, which contains pyrin domain and HIN domain (PYHIN) and absent in melanoma 2 (AIM2), has also been elaborately discussed. Members of NLR family and the PYHIN family, which can sense a wide range of pathogen‐associated molecule patterns (PAMPs) and damage‐associated molecule patterns (DAMPs) breaching cytosol, also act as the sensor. When stimuli are sensed by these “sensors,” the latter can form cytoplasmic multiprotein complexes termed “inflammasomes” with apoptosis‐associated speck‐like protein containing a caspase recruitment domain (CARD) (ASC), which is the adaptor working as bridge to connect the sensors and procaspases. When procaspases are activated and become the effector of inflammasome complex, they can act as scissors to cleave the precursors of gasdermins and proinflammatory cytokines. Maturation of pore‐forming protein gasdermins, which oligomerize to form pores in the membrane of cells or other organelles,^1^ leading to the release of these cytokines and eventually the lytic cell death. The activation of inflammasomes under certain circumstances will not definitely lead to cell death, but rather some a hyperactive state of immune cells for continuous effects.^2^, ^3^ Dysregulation of inflammasome assembly has been proved to be involved in multiple diseases, from numerous infectious diseases^4^, ^5^, ^6^, ^7^ to chronic inflammatory diseases like cancers^8^, ^9^, ^10^ and atherosclerotic cardiovascular diseases (CVDs).^11^, ^12^, ^13^, ^14^ Nevertheless, inflammasome tends to play dual roles in human diseases, depending on the cell types and microenvironment of the organs.

In recent years, with the advances in technologies and further in‐depth researches of inflammasomes in more disease models, scientists have provided us with new evidence for more precise regulation of inflammasome functions and application value of targeting inflammasome in treatment regimens. Here, we first review the activation process of inflammasomes to provide an overview insight. Then, we introduce the progress in structure of inflammasomes and three‐dimensional structure of proteins. We also introduce their biological functions under both physical and pathological conditions, which leads us to the therapeutic potential of targeting inflammasomes in human diseases.

## 2 ACTIVATION OF INFLAMMASOMES

Now known inflammasomes include NLRP1, NLRP3, NLRC4, AIM2, NLRP6, NLRP10 inflammasomes, and so on. Though the mechanisms of different inflammasomes activation have not been fully articulated so far, in this review we summarize the current understanding of the activation mechanisms of NLRP3, AIM2, and NLRC4, which are the most well researched.

### 2.1 NLRP3

**FIGURE 1:**
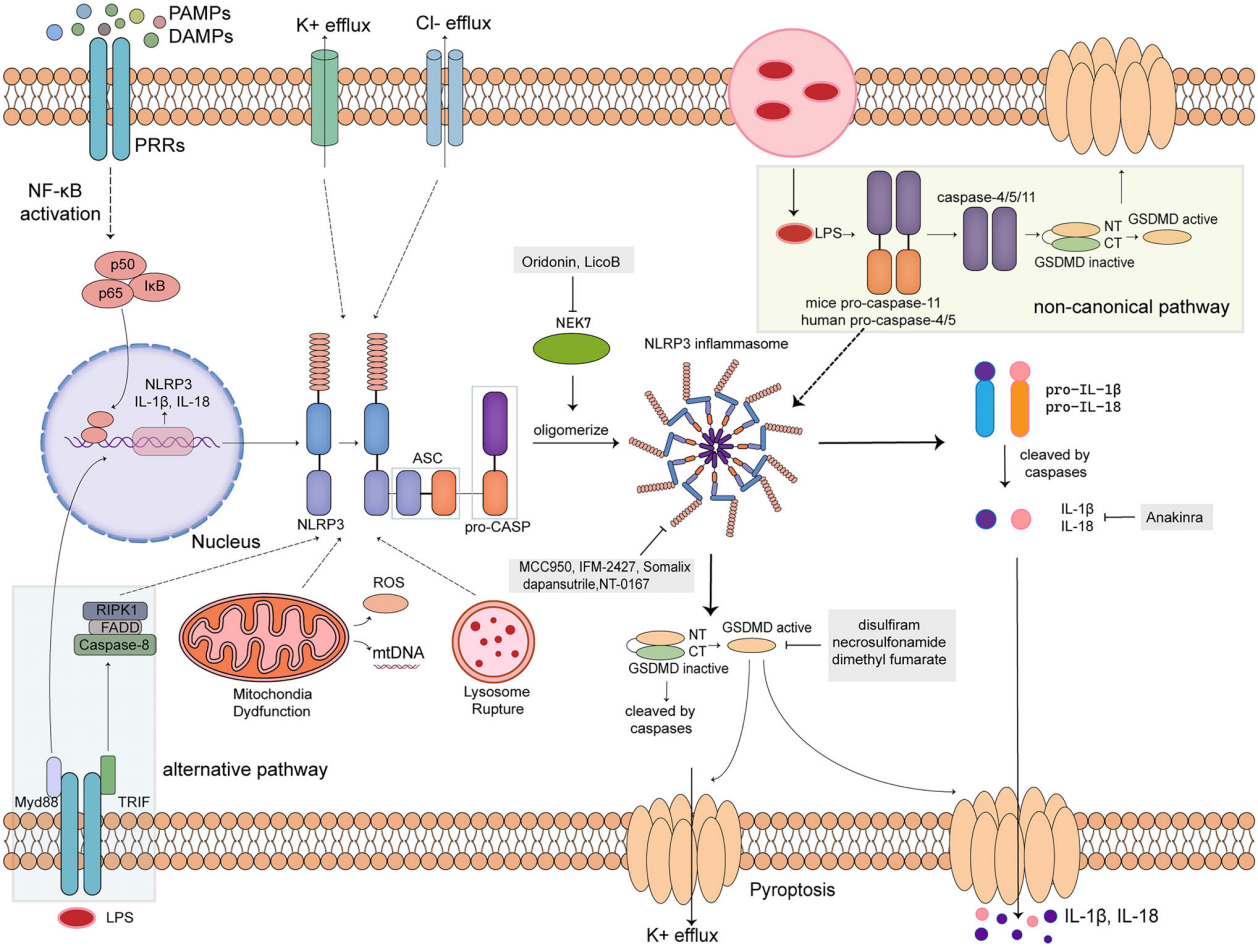
Overview of the formation and activation process of NLRP3 inflammasome. In the canonical pathway, the activation of NLRP3 is consisted of a priming and activation step. The priming step refers to the binding of PAMPs and DAMPs to PRRs and activation of NF‐κB pathway. The activation step and the assembly of NLRP3 inflammasome lead to the maturation of proinflammatory cytokines and cell pyroptosis. NLRP3 inflammasome can also be activated in noncanonical and alternative pathways.

NLRP3 is a cytosolic protein that is composed of three domains, leucine‐rich repeat (LRR) at the C‐terminal, nucleotide‐binding, and oligomerization domain NATCH and pyrin domain (PYD) at the N‐terminal.^15^ It is by far the most well‐characterized among other inflammasomes due to the fact that it can function in both innate and adaptive immune responses and acts as a bridge. The activation of NLRP3 inflammasome is a two‐step process, including a priming step and an activation step (Figure 1). As the constitutive expression level of NLRP3 is not sufficient for the activation and assembly of the inflammasome, cell can first recognize PAMPs or DAMPs with TLRs, NLRs, or other members of PRR family, resulting in the translational upregulation of *Nlrp3* and *IL‐1β* through the activation of NF‐κB pathway.^16^, ^17^ Posttranslational modifications (PTMs) including phosphorylation and deubiquitination can also happen during this step.^18^, ^19^ The next is the activation step, which can be induced by a wide variety of molecule and cellular events including changes in ion gradients and mitochondrial dysfunction.^20^, ^21^, ^22^, ^23^ For its being activated by extracellular danger signals, NLRP3 inflammasome can respond to host‐derived moieties, such as extracellular adenosine triphosphate (ATP), uric acid crystals, calcium phosphate dihydrate, cholesterol crystals, and glucose,^24^ or the stimuli of whole pathogens, such as bacteria,^25^ fungi,^26^ and viruses.^27^ It can also be activated by host‐derived molecules forming during the progression of the diseases. For instance, deposition of amyloid‐β peptide, a heterogeneous mixture of small peptides generated by sequential cleavage of amyloid precursor protein, is recognized as an important character of Alzheimer's disease (AD). It was demonstrated to function as an activator of NLRP3 inflammasome in microglia.^28^ Accumulation of damaged, ROS‐generating mitochondria caused by autophagy/ mitophagy blockade can activate NLRP3 inflammasome as well.^23^

There are three distinct ways of NLRP3 inflammasome activation (Figure 1). For the first pathway, after both the priming and activation steps, linear ASC ubiquitination with the linear ubiquitin assembly complex is indispensable for the following biological processes.^29^ NLRP3 recruits and oligomerizes with ASC that acts as the adaptor between NLRP3 and procaspase‐1 through homologous recognition of their CARD domains. Then, it can recruit and process the inactive procaspase‐1. Mature caspase‐1 acts as a scissor, cleaving both the precursors of proinflammatory cytokines and gasdermin family proteins, converting them into biological active forms, and eventually leading to the release of the mature cytokines and cell pyroptosis. This whole process is referred to as canonical inflammasome pathway and it is caspase‐1 dependent. In addition to the activation pathway described above, caspase‐4/5/11‐dependent noncanonical NLRP3 activation was also reported in recent years.^30^, ^31^ Caspase‐4/5 in human or caspase‐11 in mice can detect and then bind with lipopolysaccharide (LPS) directly in both monocytes and nonmonocytes, leading to oligomerization and autoproteolysis of the proteins.^32^ The subsequent membrane pore forming and changes in ion gradients lead to canonical NLRP3 inflammasome pathway, showing the interplay between these two pathways.^33^ Apart from the exogenous LPS, self‐encoded oxidized phospholipid 1‐palmitoyl‐2‐arachidonoyl‐sn‐glycero‐3‐phosphorylcholine (oxPAPC) in dendritic cells (DCs) can elicit this activation pathway as well.^34^ In a recent research, the nuclear orphan receptor Nur77 has been identified as a bridge between LPS and NLRP3 activation. It can bind with LPS with its C terminus directly, leading to its binding with NLRP3. It is worth noting that the binding between Nur77 and NLRP3 requires the existence of both LPS and double‐stranded DNA (dsDNA). This explains the mechanism how NLRP3 inflammasome is activated by caspase‐11 activation in response to LPS.^35^ The last way of activation is referred to as alternative NLRP3 inflammasome activation pathway, and it depends on the TLR4–TRIF–RIPK1–FADD–CASP8 signaling pathway uncoupled with other inflammasome components.^36^ In human monocytes, apolipoprotein C3 (ApoC3) was also shown to activate this caspase‐8‐dependent alternative NLRP3 inflammasome pathway through dimerization of TLR2 and TLR4.^37^, ^38^ In summary, different downstream caspases characterize different activation pathways of NLRP3 inflammasome and result in different processing methods toward the precursor of the inflammatory cytokines in macrophages and monocytes, indicate the differences in their functions. It is also worth noting that caspase‐8, the determinant of alternative activation pathway, showed both prodeath and prosurvival functions. And interactions among caspase‐8, caspase‐11, and RIPK3 navigate the cells to distinct fates,^39^ which suggests a strategy of targeting different activation pathways for disease treatment.

What attracts great attention is the mechanism for NLRP3 to sense such a wide arrange of stimuli. Researchers have discovered that much of the stimuli can lead to a novel disassembly of trans‐Golgi network (TGN).^40^ Through the ionic bonding between a region in NLRP3 and the negatively charged phosphatidylinositol 4‐phospahte (PI4P) on dTGN, NLRP3 is recruited, and utilizes dTGN as a scaffold for its aggregation. Though the precise mechanism of how these stimuli lead to the structure change of dTGN still remains to be solved, this breakthrough obviously provide insight into prevention and targeted therapy of NLRP3‐related diseases. Based on the recognition of the importance of vesicles containing PI4P in NLRP3 activation, following work in the biological characteristics of the vesicles to which NLRP3 is recruited demonstrated that they are of endosomal origin with bio‐markers include EEA1, RAB5, and SNX2.^41^ Through the observation of early endosomes with transferrin loading, researchers also found that NLRP3 is recruited to early endosomes, which accumulate both PI4P and transferrin, then the recruited NLRP3 is activated and lead to the inflammatory cascades. Taken together, these results suggest Golgi apparatus as an ideal therapeutic target of NLRP3‐related diseases.

It seems that most of the stimuli for NLRP3 inflammasome activation function through potassium (K^+^) efflux. But the precise molecule mechanisms underlying are still a biological mystery remained to be resolved. Recent studies provided us with valuable insights. Never in mitosis A‐related kinase 7 (NEK7) has been recognized as an upstream signal of NLRP3 activation.^42^, ^43^ This activation mechanism is NLRP3–NEK7 specific, as it is not observed in NLRC4 or AIM2, nor in other members of the NIMA‐related kinases family.

### 2.2 AIM2

AIM2, which belongs to the IFI family, was found in researches searching for DNA sensors and acts as a cytoplasmic DNA sensor.^44^ It was further demonstrated that the silence of *Aim2* can inhibit the activation of caspases. Among the genes of the same function, AIM2 is the only one to be able to activate caspase‐1 when stably expressed in 293T‐caspase‐1‐ASC cell line, indicating the involvement of ASC as the adaptor.^45^ Following researches suggested that AIM2 is a sensor protein that works in the similar way as NLRP3, recognizing dsDNA and oligomerizing to assemble with ASC and procaspase‐1 to form a multiprotein complex, ultimately leading to the release of mature proinflammatory cytokines and pyroptotic cell death. It was also suggested that in response to the infection of specific pathogens such as HSV and *Francisella*, AIM2 inflammasome assembly can lead to PANoptosis, which is characterized by the concomitant activation of pyroptosis, apoptosis, and necroptosis, as a protection mechanism for the host.^46^

**FIGURE 2:**
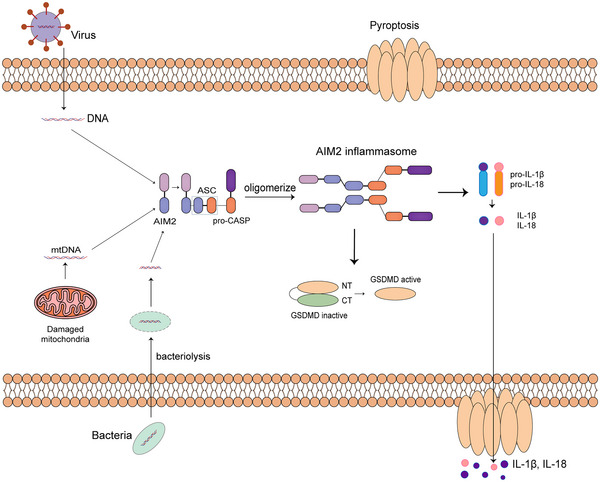
Activation process of AIM2 inflammasome. AIM2 shows high sensitivity recognizing cytoplasm DNA from inner and outer damages. After recognizing dsDNA, AIM2 oligomerize with ASC and procaspase‐1 and form protein complex. Concentration of dsDNA in cytoplasm after bacterial infection can increase through bacteriolysis.

The dsDNA upstream of AIM2 activation can originate from bacterial infection, viral infection and self‐DNA distributed in cytoplasm (Figure 2). Living bacteria including *Francisella tularensis*, *Streptococcus pneumoniae*, and *Legionella pneumophila* were proved to be able to activate AIM2 inflammasomes.^45^, ^47^, ^48^ What these bacteria have in common is that the infection processes often involve bacteriolysis to expose bacterial DNA to AIM2.

AIM2 is expressed in a relatively lower level compared with the situation with the existence of dsDNA, and there is only small chance for its oligomerization, which is important for binding with dsDNA and the interaction with ASCs. The activity of cellular AIM2 shows a significant increase after the stimuli of dsDNA in certain size.^44^ One important pathway for the dsDNA recognition and AIM2 inflammasome activation is type I interferon pathway. After *Francisella* infection, the concentration of the dsDNA is insufficient for recognition and assembly of AIM2 inflammasome, so the bacterial DNA can first activate other dsDNA sensor in cytoplasm such as cGAS and lead to production of type I interferons, triggering bacteriolysis and a mass of bacterial DNA releasing into cytoplasm for the activation of AIM2 inflammasomes.^49^, ^50^

### 2.3 NAIP/NLRC4

**FIGURE 3:**
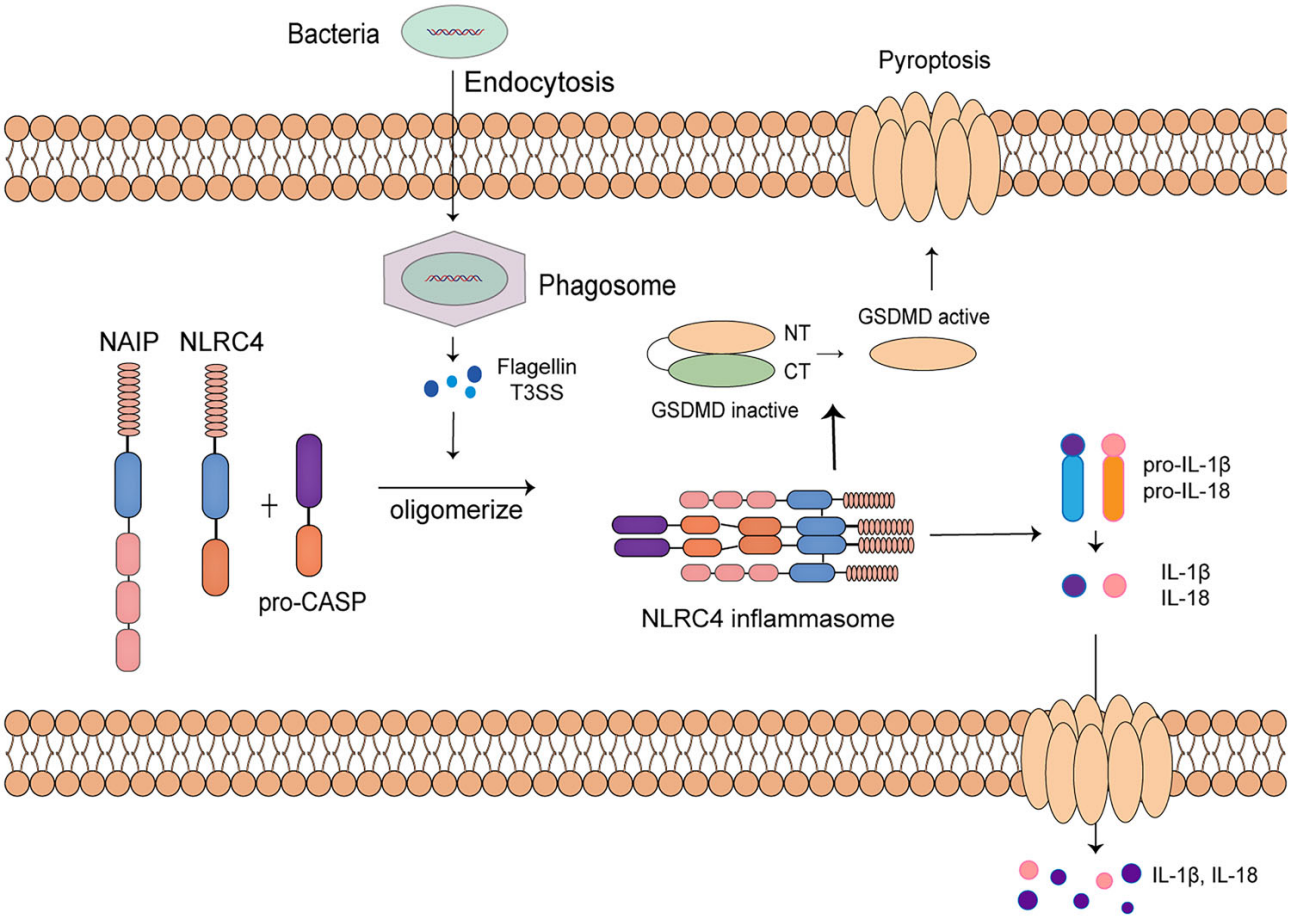
Activation process of NLRC4 inflammasome. NLRC4 activation in mice is dependent on NAIPs and responses to bacterial infections. NAIP family members act as pathogen‐sensing proteins. NLRC4 activation can be triggered when purified bacterial flagellin is delivered into cytoplasm.

Before the introduction of this part, it needs to be reminded in advance that unless otherwise specified, all NLRC4 and NLR family of apoptosis inhibitory protein (NAIP) results refer to mouse NLRC4 and NAIP, because in contrast to the observations in murine models, human cells can only encode one type of NAIP protein.^51^ Unlike NLRP3 and AIM2 inflammasome, the assembly of NLRC4 inflammasome can be facilitated by the direct recruitment of procaspase‐1 with the CARD domain of NLRC4. Some specific functions of NLRC4 inflammasome may not require the involvement of ASC, but the participation of the NAIPs. For the activation of NAIP/NLRC4 inflammasome, NAIP family members act as pathogen‐sensing proteins. NAIPs contain a Baculovirus inhibitor‐of‐apoptosis repeat (BIR) domain at the N‐terminal. Emerging evidence suggested that NAIP family proteins are involved in the NLRC4‐medaited response to pathogens. For example, NAIP5 is related to the NLRC4 oligomerization in response to flagellin, a globular protein that arranges itself to form the filament in a bacterial flagellum,^52^ while PrgJ, the bacterial rod protein, can induce NAIP2‐mediated NLRC4 oligomerization and activation.^53^ Currently, there is no evidence showing the direct interaction between NLRC4 and T3ss or flagellin. Once NAIP proteins are activated, they can interact with inactivated NLRC4 that has not yet oligomerized, leading to the recruitment of more NLRC4 monomers and their oligomerization in a self‐propagating way.^54^ (Figure 3) Apart from the activation form of binding to the protease caspase‐1 directly with the CARD domain and skipping ASC, NLRC4 can also bind to ASC, resulting in a more efficient form of inflammasome activation and the inflammatory reactions followed.

Studies of NAIP/NLRC4 inflammasome focus mainly on conditions of bacterial infection, due to the fact that quite a few gram‐negative bacteria can induce pyroptosis through NLRC4‐caspase‐1‐dependent pathway. By delivering purified bacterial flagellin into the cytoplasm, caspase‐1‐dependent NLRC4 activation can be triggered.^55^ What was observed about NAIPs/NLRC4 inflammasome first is that *Salmonella* infection in rodents can cause pyroptosis of macrophages in vitro. But in vivo systemic infection of *Salmonella* can escape from the immune detection, owing to the downregulated expression of flagellin and hiding from NAIPs/NLRC4 inflammasomes for this reason. Accordingly, the expression of flagellin can contrarily be elicited by NLRC4 for the clearance of pathogen.^56^ Recently, advances have been made in the understanding of human NAIP/NLRC4. First experiment into hNAIPs showed that T3SS needle protein but not flagellin can activate hNAIP/NLRC4 inflammasome in human cell lines.^57^ Following researches demonstrated that in human macrophages, hNAIP/NLRC4 can recognize both the T3SS needle protein and bacterial flagellin.^58^

Under hyperactivated state, NLRP4 can lead to three inflammasome‐dependent cell death pathways in strict orders. The first one is the NLRC4–caspase‐1–gasdermin D (GSDMD) pyroptosis pathway, the impairment of which can lead to the second one, NLRC4–ASC–caspase‐8 apoptosis pathway, and when both of these two pathways are blocked, the NLRC4–caspase‐1–caspase‐3 apoptosis pathway, is initiated.^59^ These research results explained the fact that during the canonical inflammasome pathway, the deletion of Casp1 can block the pyroptotic pathway but cell death can still happen in apoptosis‐dependent manner, and also demonstrated the fact that it might be necessary to block all the inflammasome‐related cell death pathways for the mitigation of corresponding inflammatory diseases.

## 3 STRUCTURE OF INFLAMMASOMES

**FIGURE 4:**
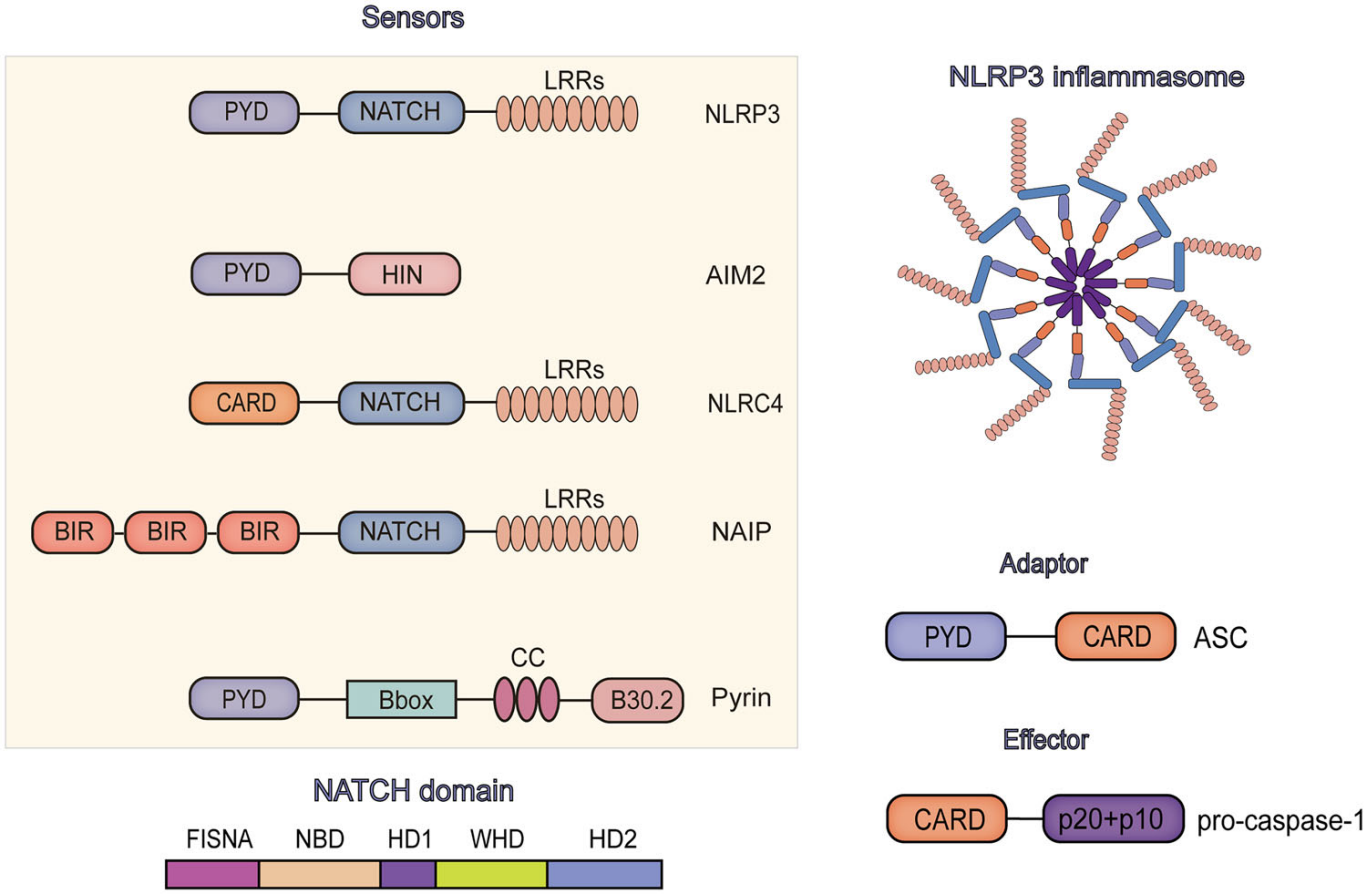
Basic structure of the sensors, adaptor and effector of inflammasomes. Schematic diagram of structure of NAIP and NATCH domain are also shown here. Sensors of inflammasome bind with the adaptor with PYD domain, while the recognition between adaptor and effector is carried out through CARD domain.

As have been mentioned, inflammasomes are multiprotein complexes and are composed of a sensor, an adaptor and the downstream effector procaspase family proteins. The early works for the structure analysis of inflammasomes were based mainly on X‐ray and nuclear magnetic resonance (NMR) technology. The basic structures of a few sensor proteins are shown in Figure 4. Recently, as scientists have made great progress in the cryo‐electron microscopy (cryo‐EM), more detailed elucidation of the three‐dimensional molecular architectures of inflammasomes are thus provided. Here, we summarize the recent advances being made in the structure analysis of several most important inflammasomes in both active and inactive forms.

### 3.1 NLRP3 inflammasome

NLRP3 inflammasome is expressed in multiple types of cells, including myeloid cells, endocrine cells, muscle cells, neurons, and so on. It is also the most mysterious one given the fact that it can be activated by a wide variety of stimuli. The structure of full‐length NLRP3 always remains as a challenge, due to the difficulty of its purification and stabilization in vitro, as well as the limitation in technologies.

NLRP3 is given the name according to three domains within the protein, the centrally located triple‐ATPase NATCH domain, PYD at the N‐terminal and LRR domain at the C terminus (Figure 4). PYD together with death domain (DD), death effector domain (DED) and CARD are members of DD superfamily A cryo‐EM study showed that NATCH domain contains the nucleotide‐binding domain (NBD) as a part, together with a helical domain 1 (HD1), a winged helical domain (WHD) and a variable helical domain 2 (HD2). And LRRs are of curved shape, resulting in an earring‐shaped conformation of NLRP3.^60^ Being supported by X‐ray crystallography and NMR techniques, it has been reported at 1.7 Å resolution that the PYD domain is equipped with the canonical antiparallel six‐helical (H1–H6) bundle structural fold of DD superfamily with five connecting loops.^61^ Residues buried within the core of NLRP3 PYD are highly conserved among different PYDs, which implies the same mechanism to interact with ASC when NLRP3 is activated and PYDs are exposed on the surface of the protein complex and are not are not shielded by NACHT‐LRR. In addition, authors have identified a disulfide bond between Cys‐8 and Cys‐108 of α1 helix (H1) and loop connecting PYD and NATCH respectively, and this bond might be important for NLRP3's being activated by ROS.^61^

A recent study using cryo‐EM technology revealed the native oligomeric form of murine NLRP3 in cells, identifying the structure of the full‐length mouse NLRP3 as double‐ring cages.^62^ They used negative‐staining electron microscopy (EM) and revealed that the inactivated monomers of NLRP3 are 20–25 nm in size. Three‐dimensional structure of the NLRP3 was then reconstructed, being stabilized by dATP at resolution of 4.2 Å and used for model building. Noticeably, the final atomic model containing NATCH and LRR domains failed to model the PYD. The NLRP3 cage is formed through “face‐to‐face” and “back‐to‐back” interfaces of LRRs, both of which are indispensable, and the NATCH domains are barely in contact with other components. The disruption of PYDs can disrupt the structure and lead to dysfunction of NLRP3, which is shielded within the NATCH–LRR cages to avoid its binding with ASCs and assembling of NLRP3 inflammasomes therefore. It was also demonstrated that this double‐ring cage structure is necessary for NLRP3's membrane binding characteristic.^62^ A following research purified full‐length, wild type human NLRP3 from baculovirus infected insect cells.^63^ To stabilize the protein, scientists added CRID3 (or MCC950), a NLRP3‐specific inhibitor during the purification procedure. Using Titan Krios microscope to collect cryo‐EM data from 1588,061 particles, they reconstructed the model of NLRP3 decamer at a resolution of 4.1 Å, as well as a focused map of the LRR and the NATCH module at a resolution of 3.9 Å. They also identified eight subdomains of NLRP3 with the atomic model, PYD (3–94), and a linker segment (95–130); the fish‐specific NATCH‐associated (FISNA) (131–218), NBD (219–372), HD1 (373–434), WHD (435–541), and HD2 (542–649) for the NATCH domain; and transition LRR (trLRR; 650−742) and canonical LRR (cnLRR; 743−1036) for the LRR domain, stretching our understanding of the NLRP3 structure.^63^

The release of NLRP3 from this autoinhibitory state requires the involvement of a series of kinases including NEK7 and phosphorylation of the upstream loop of the NBD. In a recent research, scientists coexpressed full‐length NLRP3 and NEK7 in Expi293F cells, then stimulated the cells with nigericin as the activator, activating human NLRP3–NEK7–ASC inflammasome. Then they used ATPγS, an ATP analog to stabilize the inflammasomes in the activated state. Cryo‐EM was applied to determine the structure of the complex at the resolution of 3.4 Å, showing that it is composed of 10 or 11 subunits, constituting a 10‐fold disk or a 11‐fold one. Remarkably, the binding site of NEK7 with the NLRP3 cage is competitive with the LRR–LRR interaction, indicating that NEK7 works to open the autoinhibitory cage. In contrast to the inactive state, NATCH domain rotates by ∼85.4° at the joint site of HD1 and WHD. It is worth noting that they have constructed an intact FISNA domain, and compared with inactive form, the disordered loop1 and helix2 became ordered, and conformation changes happened in loop2. These changes were demonstrated to be the prerequisite for autoactivation of NLRP3 and the formation of the oligomers.^64^

### 3.2 AIM2 inflammasome

AIM2 is composed of a PYD at the N‐terminal that interacts with ASCs and a IFN‐inducible nuclear localization (HIN) domain at the C‐terminal which is responsible for the recognition of bacterial and viral dsDNA (Figure 4).^65^ It is observed in AIM2 PYD that it can self‐oligomerize, making it hard to analyze the fine structure. Accordingly, appropriate mutation sites for deaggregation of AIM2 PYD are the key to solving the problem. Scientists chose F27 as the mutation for research and demonstrated its involvement in self‐association of AIM2 PYD.^66^ Then they set up the corresponding mutant of AIM2 PYD and used molecular replacement in the program MOLREP. The structure analysis of AIM2 PYD revealed a highly conserved six‐helical bundle structure of the DD superfamily, and the crystal structure is similar to that of NLRP3 PYD at the resolution of 1.8 Å.^67^ Let it be added here that early studies using NMR to characterize eight human PYDs and two mouse PYDs found out that all known PYDs share a characteristic short α3 helix in comparison with other DD superfamily members.^68^ Multiple cytosolic filaments can be formed by these homotypic proteins, such as the DED filaments of caspase‐8.^69^ And the PYD of AIM2 are short and dynamic, being the most variable among all the known PYDs. Scientists purified human AIM2 with it cloning into a pET30a‐derived vector with two tags, then concentrated for crystallization. X‐ray diffraction data suggested that the AIM2 PYD may bind the ASC PYD and the AIM2 HIN domains through overlapping surface.^67^

Similar to NLRP3, scientists also attach great importance to elucidate the structure detail of AIM2 in both active and inactive forms. Using X‐ray crystallography to determine the crystal structure of AIM2 HIN domains in complexes with dsDNA, it has been revealed that the nonsequence‐specific DNA recognition is achieved through electrostatic interactions between positively charged HIN domains and the sugar‐phosphate backbone of dsDNA, and AIM2 is released from its autoinhibitory state after this combination.^70^ However, only dsDNA but not ssDNA is capable of activating AIM2 inflammasome,^44^ possibly due to the binding of HIN domains to both major and minor grooves of dsDNA. Further mutagenesis studies were carried out to determine the exact residues functioning in the binding procedure. The oligonucleotide/oligosaccharide‐binding (OB) fold domains can bind with single‐stranded and play an important role in the process after DNA damage. HIN is a variation of the OB fold consisting of a tandem pair of OB folds, OB1 and OB2.^71^ The data showed that several residues such as K204A at OB1, K251A at the OB1–OB2 linker, and K309A at OB2 participate in the affinity of AMI2 HIN domains to dsDNA via hydrogen bonding, van der Waals interactions and salt bridges. Grouped mutations showed even more prominent effects. Mutagenesis experiments on full‐length AIM2 also led to impaired association between AIM2 and dsDNA, as well as the reduction of IL‐1β.^70^

Of note, binding of dsDNA to HIN domains does not deny the function of AIM2 PYD in dsDNA recognition. Fluorescence anisotropy experiments were carried out to determine the role of PYD in dsDNA binding. Results suggested that full‐length AIM2 has higher affinity with dsDNA in comparison with HIN domains alone. Adding to the fact that mutations in PYD residues can impede the binding of full‐length AIM2 with dsDNA, AIM2 PYD is admittedly involved in the dsDNA binding.^72^

### 3.3 NLRC4 inflammasome

Two domains are shared between NAIPs and NLRC4, the LRR domain and NATCH domain consists of NBD, HD1, WHD, and HD2, while NAIP also contains three BIR domains at the N terminal and for NLRC4 a CARD (Figure 4). A study reported the crystal structure of NLRC4 in closed/inactivated form demonstrated that the adenosine diphosphate (ADP)‐mediated interaction between NBD and WHD play an important role in the stabilization of NLRC4 in inactive form and its closed conformation. HD2 can contact a conserved α‐helix of NBD. These explain the autoinhibition mechanism of NAIP/NLRC4 and the function of NBD within.^73^ Scientists further disrupted the ADP‐mediated interactions between NBD and WHD or between NBD and HD2, leading to constitutive activation of NLRC4.^73^

An activated NAIP can recognize and bind with NLRC4 for the formation of inflammasome via ligand recognition.^74^ NAIPs can at the same time bind with conserved ligands of bacteria, then recruit NLRC4 as the adaptor for the assembly of functional inflammasomes. NAIP2 can detect the inner rod protein of bacterial type III secretion system, while NAIP5 and NAIP6 can detect bacterial flagellin, in response to which was NLRC4 first observed.^55^ A research assembled the FliC‐activated NAIP5‐NLRC4 complex and the PrgJ‐activated NAIP2‐NLRC4, then purified the proteins. EM data suggested that there is only one single NAIP exists in each complex, whether in a full disk or a partially assembled disk.^75^ Cryo‐EM data on PrgJ–NAIP2–NLRC4 complex suggested that an inflammasome disk is composed of an inner ring and an outer ring from the top or bottom view with 11 or 12 copies of NAIP2 or NLRC4. The inner ring contains the NBD, HD1, and the WHD, while the outer ring consists of HD2 and the LRR domain.^75^ It was observed in apoptosome assembly by Apaf‐1 that the conformation transition is achieved through exchange of ADP in the inactive state to ATP in the active state.^76^ Authors tested if the same mechanism exists in the conformation transition of NLRC4, and the results indicated that there may exist alternative mechanisms. The NAIP1–NLRC4 interactions were further analyzed and it was shown that the NAIP2–NLRC4 interactions are extensive with a total surface area of ∼1000 Å^2^ per subunits per interaction, and contain a mixture of hydrophobic, hydrophilic, and charged interactions.^75^

A study in 2017 using cryo‐EM reported the structure of the assembled ∼1.4‐megadalton flagellin–NAIP5–NLRC4 inflammasome, revealing that six distinct NAIP5 domains contact multiple conserved regions of flagellin, presenting NAIP5 into an open and active conformation for the assembly and activation of inflammasomes.^77^ A following study in 2018 reported the cryo‐EM structure of NAIP5–NLRC4 at the resolution of 4.28 Å. For the stabilization of the activated form of NAIP5–NLRC4, the flagellin derivative for interacting with NAIP5 domains can be buried after its binding with NAIP5.^78^ Both studies suggested that flagellin can bind to a deep pocket‐like structure formed by domains of NAIP5.

Most studies of NAIP/NLRC4 structure focus on murine NAIPs, as little is known about NAIPs in other species, especially human NAIPs. Recent studies with whole exome sequencing found out that human encode only one copy of full‐length NAIP protein, with some other truncated ones. We speculate that it is due to the high selective pressure for the organisms to survival under hyperinflammatory conditions. It has been reported that among seven mouse NAIPs, it is NAIP1 that functions as the mouse counterpart of hNAIP,^57^ which may indicate the similarity in their structure.

### 3.4 Pyrin inflammasome

Pyrin inflammasome was discovered in researches of the molecular mechanism of familial Mediterranean fever (FMF) and pyrin‐associated autoinflammation with neutrophilic dermatosis,^79^ and was demonstrated to be one of PRRs as an innate immune sensor. Once being activated, pyrin can assemble into protein complex with other components of inflammasome, leading to release of IL‐1β, IL‐18, and cell pyroptosis.^80^, ^81^ Pyrin is localized mainly in immune cells, and its expression can be regulated by various proinflammatory cytokines and pathogen components.^82^, ^83^, ^84^

Human pyrin, which is a member of the superfamily of tripartite motif‐containing (TRIM), contains four functional subunits, that is the PYD, a zinc finger domain (Bbox), a coiled coil (CC) domain, and a B30.2/SPRY domain, while the pyrin of mice lack the B30.2/SPRY domain (Figure 4).^85^ But the lack of ring domain makes pyrin an incomplete member of TRIM family. Report also proposed that pyrin oligomerizes via the CC domain.^86^ Coimmunoprecipitation experiments indicated the function of B30.2/SPRY domain when overexpressed to interact with other inflammasome such as NLRP1, NLRP2, and NLRP3 as well as caspase family proteins, caspase‐1, and caspase‐4,^87^, ^88^ suggesting that pyrin inflammasome might be capable of regulating the function of other inflammasomes. Pyrin only shares the similarity of the PYD domain in structure with other sensors of inflammasome such as NLRP1, NLRP3, and AIM2, for the important role of PYD domain to interact with ASC.^89^ This is consistent with the theory of structure and function adaptation.

### 3.5 Structure of other components of inflammasome complex

#### 3.5.1 ASC

ASC consisting of an N‐terminal PYD (amino acid M1–T89) and a C‐terminal CARD (amino acids H113–S195) is a ∼24 kDa cytosolic adaptor protein of relatively conserved sequence.^90^, ^91^ It is expressed mainly in nucleus without stimuli, but translocate to cytoplasm and assembles into ASC specks after stimulation.^92^ Then it can bind with both the sensors and procaspases with PYD and CARD domains respectively through homotypic interaction, leading to the formation of an activated and functional inflammasome protein complex.^90^, ^93^ Both PYD and CARD of ASC possess a canonical six‐helical bundle motif fold, while the former showing a PYD‐specific feature of a long loop between helices α2 and α3, and the helix 1 of the latter is absent with H1a.^91^

For the activation of inflammasomes, ASC can form large oligomeric filaments to facilitate the recruitment of procaspases.^94^ Sborgi et al.^95^ applied cryo‐EM and NMR and determined that ASC assembles to long filaments in response to pathogen invasion and thus amplifies the inflammatory signal. The PYD of ASC forms the helical filament core while the CARD is flexibly attached to the filament periphery.^96^

#### 3.5.2 Caspases‐1

Caspase‐1, an inflammatory cytokine converting enzyme, was initially identified as the protease responsible for maturation of pro‐IL‐1β, and belongs to the aspartate‐specific cysteine protease family.^97^ Procaspase‐1 is the inactive form of caspase‐1, and is expressed as a 404 amino acid‐monomer and catalytically dormant tripartite proenzyme. Procaspase‐1 consists of one N‐terminal CARD and a catalytic domain being composed of subunits p20 and p10, and is activated through autoproteolysis regulated by the assembly of inflammasomes or ASC pyroptosome. According to recent studies, CARD–CARD interaction between ASC and procaspase‐1 facilitates the recruitment of full‐length procaspase‐1 to inflammasome, generating an active form which can self‐process into mature caspase‐1 through cleavage of the link between p20 and p10 subunits. Procaspase‐1 can also be cleaved and activated by caspases in an amplification cascade manner. Generation of active caspase‐1 leads to cleavage of pro‐IL‐β, pro‐IL‐18, and inactive gasdermin family proteins, triggering proinflammatory cytokine release and pyroptotic cell death.

The CARD of caspase‐1 alone can polymerize into a filamentous macrostructure consisted of multiple subunits, with the inner diameter of ∼10 Å and the outer diameter of ∼80 Å, respectively. And the configuration is similar to that of the Myddosome DD complex and the MAVS CARD filament.^98^ In this mentioned study, the caspase‐1 CARD domain filament was reconstructed with cryo‐EM technology, and structure was determined therefore. There are three types of asymmetric interactions between three interfaces. Site‐directed mutants were generated to validate the importance of residues of these interfaces.^98^ Another research suggested that PYD and CARD of caspase‐1 both form filaments. In the presence of full‐length ASC or ASC CARD, polymerization of caspase‐1 CARD can increase as a result of activation of AIM2 or NLRP3.^96^

## 4 BIOLOGICAL FUNCTIONS OF INFLAMMASOMES

### 4.1 Overview of biological functions of inflammasomes

**FIGURE 5:**
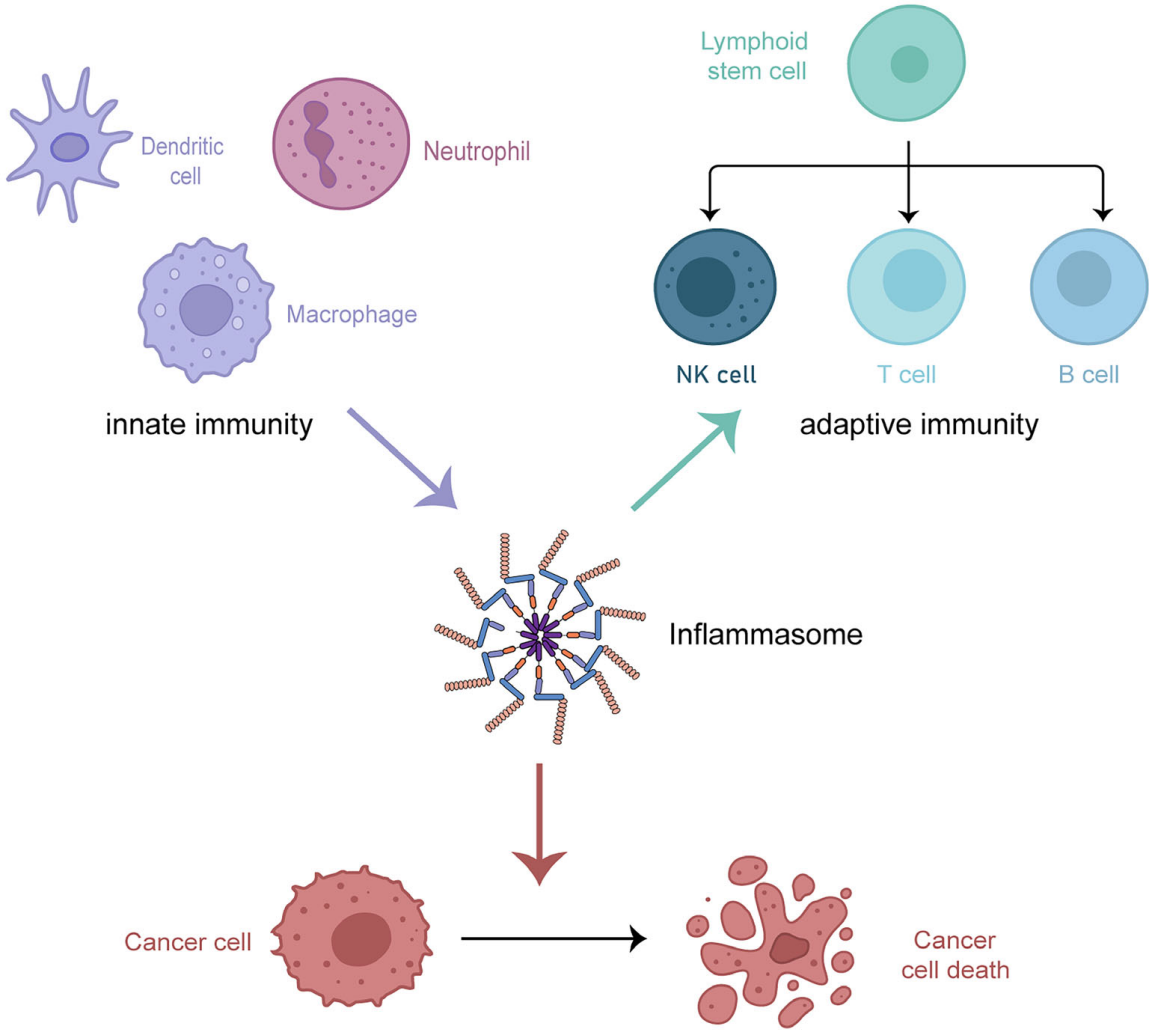
Inflammasomes work as a bridge between innate and adaptive immunity in immunotherapy for tumors. Inflammasomes play an important role in detection of pathogens in innate immune cells, presenting these danger signals to adaptive immune cells. Inflammasomes also contribute to the development of adaptive immune cells. In tumor‐associated inflammation (TAI), inflammasomes can result in cancer cell death. This figure is created with BioRender.com.

The mobilization of immune system in mammals involves two distinct and interrelated systems, the innate immunity and adaptive immunity. The antigen‐presenting cells including macrophages, DCs, and so forth function as deliverers to present the information of endogenous danger signals and exogenous pathogen irritants to the B and T lymphocytes, playing leading roles in innate immunity.^99^ For the detection of pathogens in innate immune cells, there exists two major classes of receptors, known as PRRs. One that found in the plasma membrane and endosomes can detect PAMPs and DAMPs in the extracellular environment, and this category contains TLRs and C‐type lectin receptors. Another was found to reside within the cells for detection of danger signals in the intracellular milieu, including RLRs and NLRs.^100^ Inflammasome, which is expressed in various types of cells and reacts to a wide range of physiological and pathogenic stimuli, can thus act as a bridge between innate and adaptive immunity (Figure 5). The release of proinflammatory cytokines and pyroptotic cell death will ultimately lead to the recruitment of more immune cells into the injury sites, clearing the foreign objects and repairing the tissues, or otherwise cause lingering of the disease. It was also reported that inflammasomes contribute to the development of adaptive immune cells.^101^ The ablation of *Nlrp3* in mice can damnify bone marrow B cell development and distort expression of marginal zone B cells in the spleen and B‐1a cells in the peritoneal cavity.^101^ Though researches into inflammasomes focus mainly on immune cells, they are widely expressed in other cell types as well. Epithelial cells (ECs), for instance, can secrete proinflammatory cytokines Il‐1β and IL‐18 in a NLRP3 and caspase‐1‐dependent manner with presence of *S. aureus* and extracellular ATP.^102^

The basic and prominent functions of inflammasome activation are the maturation of caspase and as a result, the secretion of proinflammatory IL‐1β and IL‐18 and cell pyroptosis. Il‐1β and IL‐18 both belong to the IL‐1 family. This cytokine family have a giant role influencing the immune system as they share similar functions with TLR family. These cytokines function through the interaction with their receptors, triggering innate inflammation, and can also function as DAMPs.^103^ Pyroptosis was described by Brennan et al.^104^ in a research of *Salmonella*‐induced macrophage cell death. By applying DNA dyes to mark rupture of cells, they observed a rapid and lytic cell death after infection. Due to cognitive limitations, this phenomenon was mistakenly described as a form of apoptotic cell death caused basically by bacterial virulence. But what this actually pointed out is the biological function of inflammasome in defending against pathogen infections, which has been described in many following researches. Currently understanding of pyroptosis evolves with the discovery of canonical and noncanonical inflammasome pathways, and the downstream effector molecules they have in common, the gasdermin family proteins. In the past decade, pyroptosis has been proved to play a protective role and maintain the homeostasis of immune system, although it has also been linked with tissue damage, organ failure and lethality in sepsis when aberrantly activated.^105^, ^106^, ^107^, ^108^ In recent years, the construction of mice models by knocking out different components of inflammasome complex demonstrated that inflammasomes play central roles in the immune responses against pathogen infections. As a concrete manifestation that ASC^−/−^ were more susceptible to *S. pneumoniae* infection than wild‐type mice,^109^ and that AIM2‐deficient mice displayed an increased susceptibility to mucosal infection with *Salmonella typhimurium*, showing more severe body weight loss, intestinal damage, intestinal inflammation, and disruption of basal and activated EC turnover.^110^ Murine neutrophils, the most abundant type of granulocytes and the most abundant type of white blood cells, can express functional cell surface P2X_7_R during *S. pneumoniae* corneal infection, leading to NLRP3 inflammasome activation and IL‐1β secretion for bacterial clearance.^111^

More importantly, the protective effects of inflammasomes come into play not only in the form of pyroptotic cell death, as inflammasome activation can lead to distinct‐different cell fates.^112^ Emerging evidence also suggests that the activation of inflammasomes will not necessarily result in pyroptosis. For example, as we have mentioned, AIM2 inflammasome can fuel PANoptosis by forming a large multiprotein complex with pyrin, Z‐DNA binding protein 1 and ASC, caspase‐1, caspase‐8, RIPK3, RIPK1, FADD. This protein complex is referred to as AIM2 PANoptosome, being formed and functioning as part of the host defence.^46^ CAPS model mice carrying an *Nlrp3* ^R258w^ mutation are strongly resistant to experimental colitis and colorectal cancer through remodeling of gut microbiota with enhanced anti‐inflammatory capacity of regulatory T cells (T_regs_).^113^ NLRC4 inflammasome in neutrophils can selective promote the maturation of IL‐1β without the occurrence of pyroptosis during acute *Salmonella* infection, which is different from the condition in macrophages under the same challenge.^114^ Interestingly, inflammasomes in DCs can release inflammatory cytokines from either living (hyperactive form) or dead (pyroptotic) cells,^34^ it was reported that when encountered with bacterial components and self‐encoded oxPAPC, DCs can enter a hyperactive state, producing inflammatory cytokines through caspase‐11‐dependent noncanonical inflammasome pathway and inducing adaptive immune responses.^34^ And the release of inflammatory cytokines from living cells without cell rupture was also observed in other phagocytes.^36^, ^114^, ^115^, ^116^, ^117^ Inflammasome activators such as bacteria and other pathogen products or host‐derived oxidized lipids can lead to GSDMD‐dependent IL‐1β release from hyperactive macrophage.^116^ This also indicates diverse functions of membrane pore formed by gasdermin family proteins. It is reasonable to speculate that the aim of this hyperactive state is for the maintenance of continuously active immune reactions and thorough clearance of pathogens. There is also hypothesis that these phagocytes undergo membrane repair by endosomal sorting complexes required for transport (ESCRT) to remove the GSDMD pores, which is already been proven in pyroptotic BMDMs, but not in hyperactive cells yet.^118^

Tumor‐associated inflammation (TAI) is an important hallmark of nearly all cancers, and has the function of either promoting tumorigenesis and progression or suppressing it,^119^ which is consistent with the fact that inflammasome can play a dual role in the pathogenesis of cancer.^120^ It was back to 19th century that Rudolf Virchow, a pathologist form Germany observed infiltration of a large number of inflammatory cells in the tumor tissues, hypothesizing that tumors were originated from chronic inflammatory responses. The inflammatory cytokines produced by inflammasome activation contribute to systemic low‐grade inflammation, and forming inflammatory microenvironment in autocrine or paracrine manners.^121^ In addition, the recruitment of immune cells and fatty acid‐induced inflammasome activation's interference of insulin signaling^24^ also promote the progression of cancer. For the antitumor immunity regulated by inflammasome activation, immunogenic cancer cell death can apparently enhance antitumor immunity.^122^ There are also studies supporting the theory that inflammatory cytokines secreted by immune cells with inflammasome activated can regulate the activity of T cells, as IL −1β functions as a pivotal regulator of the T cell fates. IL‐1 administration in cells can result in enhancement of granzyme B and increased capacity for IFN‐γ production. In vivo experiments show enhancement of protective value of weak immunogens.^123^ In liver and lungs, IL −1β can lead to increase of CD4 and CD8 cells in number, and augmented differentiation of the antigen‐triggered T cells.^124^ In particular, hyperactive DCs can promote the generation of potent cytotoxic CD8 T cell immunity. In an in vitro model of interaction between DCs and T cells, bone marrow‐derived DCs showed weak capacity to active naïve or memory CD8 T cells.^2^ T cells primed by living DCs in lymph nodes, however, make a transition into a third phase of high motility and rapid proliferation.^125^ The efficacy of chemotherapy depends partly on DCs for its presenting antigens from tumor cells to T lymphocytes. Chemotherapy was also proven to promise great result when combined with the modification of inflammasomes in DCs.^126^ Oxaliplatin, doxorubicin, and other platinum‐based chemotherapeutic agents, are capable of eliciting tumor cell death in a caspase‐1‐dependent manner suggested by *Casp1^−/−^* mice model.^127^, ^128^ For another, the debris of tumor cells including ATP and HMGB1 act as DAMPs, triggering activating NLRP3 inflammasomes.^129^ Hyperactivation of the PI3K–AKT pathway caused by intrinsic loss of PTEN, a tumor suppressor, can lead to tumor resistance to anticancer therapies.^130^, ^131^, ^132^ And it has been demonstrated that PTEN expression in myeloid cells is determinant for chemotherapy‐induced NLRP3 inflammasome activation and its antitumor effect.^126^ For more accurate description of the role of hyperactive DCs and inflammasomes in antitumor therapeutics, more studies in this field are needed.

Though different inflammasomes are activated through relative separated pathways, recent evidence demonstrated that more complex interactions between inflammasomes can work in either synergy or antagonism ways. Experimental autoimmune uveitis, an experimental autoimmune disease, has been proved to associate with NLRP3 inflammasome.^133^ In the Nlrp3−/− mice, the deficiency of Nlrp3 results in upregulated transcription of Aim2, while overexpression of Nlrp3 an attenuate inflammation through inhibition of AIM2 inflammasome.^134^ This provides us with important evidence of antagonism effects among inflammasomes.

### 4.2 Functions of NLRP3 inflammasome

The biological functions of NLRP3 protein include its involvement in inflammasome formation, actions in response to oxidative stress and functioning as a transcription factor.^135^ And NLRP3 inflammasome is critical in the maintenance of homeostasis against pathogen infections and plays a dual role, as the secretion of inflammatory cytokines and cell pyroptosis can cause tissue damage at the same time of pathogen clearance.^136^, ^137^ The activation of NLRP3 inflammasome can also lead to inflammatory cell death not only depends on IL‐1, IL‐18, or pyroptosis, but rather in form of PANoptosis, a more complex integration of pyroptosis, apoptosis and necroptosis.^138^ Autoimmune disorders, of which rheumatoid arthritis (RA), systemic lupus erythematosus (SLE), inflammatory bowel disease (IBDs), and so on are the most commonly seen, show strong connection with NLRP3 inflammasome.^139^ For example, high level mRNA of NLRP3 and other components of the inflammasome complex were detected in samples from patients with RA.^140^, ^141^ It has also been demonstrated by animal models that synovial succinate accumulation is to blame for fibrosis in RA by inducing NLRP3 inflammasome activation. It has also been demonstrated that mutation of NLRP3 and CARD8 can lead to higher disease activity of RA.^142^ In recent years, scientists showed great interest in the involvement of NLRP3 inflammasome in both antitumor therapies and tumorigenesis studies. For example, it has been demonstrated that by blocking NLRP3 inflammasome activation with MCC950, antitumor immune responses in head and neck squamous cell carcinoma were improved.^143^ Recently, a study to illustrate the mechanism of *Salmonella* immunotherapy suggested the necessity of NLRP3 engagement as well as the recruitment of macrophages to tumor microenvironment, supporting the position participation of NLRP3 inflammasome in antitumor activity.^144^

### 4.3 Functions of AIM2 inflammasome

As is indicated by its activation mechanism, AIM2 inflammasome is important for protection of the body from viral infection. Some viruses even developed strategies to escape from detection of AIM2 inflammasome. For example, HSV‐1 was demonstrated to express VP22, a tegument protein, thus inhibit the AIM2 inflammasome activation and the following release of inflammatory cytokines.^145^ Interestingly, in addition to its activation through recognition of dsDNA, studies also reported the role of AIM2 inflammasome in RNA viral infection. With *Aim2* silenced, human dermal fibroblasts infected with Chikungunya virus showed reduced cleavage and maturation of caspase‐1 as well as the release of IL‐1β.^146^ But the precise mechanism for AIM2 responding to RNA viral infection requires further researches.

Abnormal expression of AIM2 in immune cells also contributes the pathogenesis of autoimmune diseases. In AIM2/DNase II dual‐deficient mice, for example, improvement of pathological manifestations of RA and reduced level of caspase‐1 and IL‐1β production were both observed.^147^ SLE also shows correlation with AIM2 inflammasome. Scientists detected the expression level of AIM2 in patients with SLE and observed an enhancement of expression in both peripheral blood and skin lesions. It was further demonstrated that AIM2 can promote T cell differentiation during SLE progression.^148^

### 4.4 Functions of NLRC4 inflammasome

As it can be activated by T3SS proteins from gram‐negative bacteria such as *Salmonella* Typhimurium (*S*. Typhimurium), NAIP/NLRC4 inflammasome is important for immune responses against gram‐negative pathogens.^114^, ^149^, ^150^ It is worth noting that *Nlrc4* knockout mice have no difference in bacterial load compared with wild type mice after *S*. Typhimurium infection, while *Nlrc4* and *Nlrp3* double knockout animals are highly susceptible to the infection. Synergy and complement in functions of each other and redundant roles for inflammasome receptors might be the reasons. NLRC4 inflammasome exists not only in immune cells, it can also assemble in intestinal ECs for protection against enteric pathogen.^151^ Research about *Helicobacter pylori* infection showed opposite role of NLRC4 inflammasome, while Nlrc4‐deficient mice control the bacterial burden better than wild type animals.^152^ This suggest that the precise function of NAIP/NLRC 4 inflammasome is pathogen specific.

Similar to other inflammasomes, mutations in NAIPs or NLRC4 contribute to autoinflammatory diseases and cancer in human as well.^54^, ^153^, ^154^, ^155^, ^156^ Macrophage activation syndrome (MAS) belongs to a broader category named hemophagocytic lymphohistiocytosis and is developed in patients with rheumatic disorders. It has been demonstrated that MAS is associated with NLRC4 mutation for the increase of IL‐18 production.^157^ By construction of NLRC4^−/−^ mice model, it was demonstrated that the knockout of *Nlrc4* leads to more aggressive tumor invasion and increased tumor numbers and load, suggesting the protective effect of NLRC4 inflammasome on colonic tumor.^158^ Another research suggested that NLRC4 can protect mice against mucosal and systemic challenges, including epithelial injury induced by dextran sulfate sodium (DSS).^159^

Apart from pyroptotic cell death, NAIP/NLRC4 inflammasome is also shown to be related to other cell death forms, such as PANoptosis. PANoptosis is regulated by PANoptosome, a molecule scaffold for the engagement of molecule components of other regulated cell death forms including apoptosis, pyroptosis, and necroptosis.^160^ While most of the studies on PANoptosis are focused on NLRP3 and AIM2 inflammasomes, evidence also suggests that NLRC4 inflammasome might also plays a part. Both the activation of PANoptosis and NAIP/NLRC4 inflammasome were observed during *S*. Typhimurium infection.^114^, ^160^ To elucidate the association and interplay between NAIP/NLRC4 inflammasome and PANoptosis, further experiments are indispensable.

### 4.5 Functions of pyrin inflammasome

As we have discussed, FMF originates from the gain of function mutation of *MEFV* gene. Treatment of the disease focuses mainly on preventing the outbreak of inflammatory responses, and administration of colchicine for inhibition of inflammation together with Anakinra in case of colchicine resistance promises great results.^161^, ^162^ Previous understanding of mutations' function in diseases can be summarized as the loss of negative regulation on other inflammasomes and the enhanced inflammation thereafter.^163^ However, there is also evidence suggesting that the gain of function mutation might explain inflammatory phenotype in FMF.^164^ Further researches for clarification are therefore needed.

Apart from the function of PYD in autoinflammatory diseases, it has also been suggested that pyrin inflammasome contributes to the pathogen pattern recognition of mammalian innate immunity in cytosol.^165^ This study elucidated that pyrin inflammasome can sense the modification of host Rho protein, thereby achieving recognition of related pathogenic bacteria. In addition, evidence for development of vaccine and neutralizing antibodies targeting TcdA/B toxin is thus provided.

### 4.6 Functions of noncanonical inflammasome

In particular, the cytoplasmic LPS of gram‐negative have been shown to directly bind with caspase‐4/5^166^, ^167^ of human or caspase‐11^168^, ^169^ of mice in the cytosol and activate noncanonical inflammasome pathway. LPS internalized through the secretion of outer membrane vesicles from gram‐negative bacteria can also trigger caspase‐11‐dependent inflammatory reaction and pyroptosis.^169^ Caspase‐11 is expressed in ECs. In the model of pulmonary ECs transfected with FITC‐labeled LPS in lipofectamine‐based liposomes, it was demonstrated that caspase‐11‐mediated pyroptosis activated with LPS can trigger cardiovascular inflammation, and can be a promising target for acute lung injury therapeutics.^170^ Here, we review the function of noncanonical inflammasome pathway focusing mainly on its role in antimicrobial defense and inflammatory responses as well as its function in some autoimmune diseases.

Intracellular LPS‐induced noncanonical inflammasome pathway was demonstrated to be caspase‐1‐dependent, implying that the stimuli will also lead to the activation of canonical inflammasome pathway simultaneously.^30^ In this mentioned research, macrophages were infected with *Escherichia coli*, *Citrobacter rodentium*, or *Vibrio cholerae*, while *Casp11^−/−^* mice showed defects in IL‐1 production. By comparison between Casp1^−/−^ and Casp1/11^−/−^ knockout mice, authors drew the conclusion that it might be caspase‐11 rather than caspase‐1 is to blame for the deleterious inflammatory responses^30^ As we have discussed, oxPAPC can elicit inflammatory cytokine production and release from living DCs.^2^ In noncanonical inflammasome pathway, oxPAPC seems to play dual roles. In macrophages, it was shown that oxPAPC can inhibit the activation of caspase‐4/11‐dependent inflammasome by competitively binding with caspases with LPS, which is consistent with the in vivo experiment that oxPAPC inhibits the noncanonical inflammasome in macrophages and protect mice model from septic shock.^171^ In DCs, however, oxPAPC activate noncanonical inflammasome that process IL‐1β without resulting in pyroptosis.^34^ To elucidate the anti‐ and proinflammatory effects of noncanonical inflammasome in pathogen infections, more researches are urgently needed to shed light on this issue.

In autoimmune diseases, the functions of noncanonical inflammasome provide us with evidence for targeted therapy as well. Multiple sclerosis (MS) is a potentially disabling disease of the brain and spinal cord (central nervous system), which has been reported to be highly relevant to canonical inflammasome activation pathway. And it has been demonstrated that noncanonical inflammasome also play a part in the progression of MS. During this disease, the immune system attacks the protective sheath that covers nerve fibers, leading to neuroinflammation.^172^ Oligodendrocytes (OLGs) are a type of neuroglia, the death of which is a major risk factor of pathogenesis of MS. And it has been demonstrated that both caspase‐1 and caspase‐11 are actively involved in OLG death. Further experiments using encephalomyelitis (EAE), an animal model of MS, suggested that the symptoms of the disease were improved in the caspase‐11‐deficient mice.^173^ In DCs where the actions of noncanonical inflammasome are well characterized, it has been reported that IFN‐β, which decreases relapse rate and disease activity of MS, can elicit apoptotic cell death of DCs by increasing the expression of caspase‐11.^174^ To sum up, caspase‐11‐dependent noncanonical inflammasome pathway can be a novel therapeutic target for MS. Diabetic nephropathy (DN) also belong to autoimmune diseases, and is characterized by sterile inflammation with the loss of renal inherent parenchyma cells, podocytes.^175^, ^176^, ^177^ Cheng et al.^178^ demonstrated that caspase‐11/4‐dependent inflammasome activation and the following GSDMD‐dependent pyroptosis contribute to podocyte loss, thus play a part in DN pathogenesis. Monosodium urate crystal (MSU) acts as a stimuli for inflammasome activation.^179^, ^180^, ^181^ Accumulation of MSU in joint spaces and chronic inflammation brought about lead to gout.^182^, ^183^ In caspase‐11‐deficient mice and macrophages derived from the animals, gout specific cytokine release was shown to decrease, and neutrophil failed to produce neutrophil extracellular traps when treated with MSU,^184^ providing evidence for targeting noncanonical inflammasome for the treatment of gout. The function of noncanonical inflammasome in IBDs has been reviewed.^185^ Authors proposed the either aggravating^186^, ^187^ or protective^188^, ^189^, ^190^ role of capase‐11 noncanonical inflammasome in the pathogenesis of IBDs.

## 5 THERAPEUTIC TARGETS OF INFLAMMASOMES

### 5.1 Inflammasome‐related diseases

According to the biological functions, we can conclude that inflammasomes have different roles under different conditions, functioning as a double‐edged sword in human diseases. Depending on whether it is related to infection, these diseases can be divided into two main categories, microbial infectious diseases and sterile inflammatory diseases.

NLRP3 inflammasome, which is the most widely studied, has been proved to play an important role in the immune responses against bacteria, virus, fungi and parasite infections mainly through indirect recognition. Quite a few studies suggest that microbe activate inflammasomes through induction of cellular stress and injury‐related signals. For example, elder mice were demonstrated to be notably more susceptible to respiratory viral infection, showing immoderate inflammation and increased mortality due to aberrant activation of STING and NLRP3 inflammasome modulated by shortened telomeres.^191^ Scientists established the animal model by exposing wild‐type mice and Terc^−/−^ mice, an animal model characterized by premature aging phenotype, to influenza A virus (IAV) and vesicular stomatitis virus, respectively. The macrophages of Terc^−/−^ mice show STING‐driven inflammatory property, leading to more severe inflammatory response and worsen survival rate compared with wild‐type mice. Ulteriorly, they detected the induction of NLRP3, cleavage of caspase‐1 and IL‐1β, and the oligomerization of ASC. The results showed that the activation of NLRP3 inflammasome was enhanced after IVA infection in Terc^−/−^ macrophages. Further experiment confirmed the hypothesis that this activation is integral into the STING‐driven pathway.^191^ An earlier research suggested that metformin can improve LPS‐induced ARDS through suppression of NLRP3 inflammasome.^192^ To avoid overactivation of inflammasome, many microbe have evolved with a system to escape from the detection of the sensor of inflammasome. For example, NS1 protein of 2009 pandemic IVA was proved to target NLRP3 inflammasome directly and suppress its activation.^193^ Since the end of 2019, severe acute respiratory syndrome coronavirus 2 (SARS‐CoV‐2) infection has been attracting great attention as it cause threat to global health. Emerging researches suggested that NLRP3 inflammasome activation is closely related to severity of coronavirus disease 2019 (COVID‐19). After the virus's entry into epithelial or endothelial cells of the lung, SARS‐CoV‐2 initiates. Released proinflammatory cytokines lead to NLRP3 inflammasome activation in infiltrated macrophages and neutrophils, resulted in hyperinflammation.^194^ In a recent research, it has been further illuminated that infected ECs produce DNA as DAMPs, which acts as the signal II in inflammasome activation in myeloid cells, leading to IL‐1β release, and eventually IL‐6 release from both ECs and leukocytes. This epithelial‐immune circuit causes inflammatory cytokine storm in severe COVID‐19.^195^ The outcome of these infectious diseases varies, and it can be either good or bad depending on the pathogens, the specific organs and tissues, the effector cells involved and the genetic background.

In sterile inflammatory diseases including crystal deposition disorder, neurodegenerative diseases and depression, atherosclerosis, insulin resistance, IBD, and cancer, the role of inflammasome is more diverse. In general, the pathogenesis of these diseases is related to the exquisite activation of inflammasomes. The activation of NLRP3 inflammasome and the following maturation and release of inflammatory cytokines are demonstrated to be important risk factors in CVDs. The CANTOS trials are a series of experiments focusing on the anti‐inflammatory thrombosis outcome of Canakinumab, a monoclonal antibody of IL‐1β. Being randomized, double‐blinded, placebo‐controlled, the trials demonstrated that the activation of inflammasome acts as the cause rather than the consequence of atherothrombosis, indicating the clinical use of this category of chemical compound in CVDs. In recent years, the researches into inflammasome in cancers have also grabbed a lot attention. Scientists attach great importance to the utilization of inflammasome modification in immunotherapy for tumors. Tumorigenesis, the proliferation of tumor cells and metastasis are all related to the alteration effect on tumor microenvironment of inflammasome.^196^ But under some specific conditions, inflammasome can work as the enemy of tumor cells.^3^

### 5.2 Advances in inflammasome‐targeted therapy

Based on the fact that the pathogenesis of many diseases is related to aberrant activation of inflammasome or unwonted genetic background, inflammasome inhibition can be an effective measure for treatment. For the inhibition of inflammasome activation and the following biological effects, current strategies include targeting the components of inflammasome complex or targeting the downstream inflammatory cytokines. For example, recent researches targeting IL‐1, the major and foremost inflammatory cytokine processed by inflammasomes, have shown great results in multiple clinical trials (Table 1). Apart from Canakinumab in CANTOS trials, other blockers including Anakinra, Gevokizumab, and Rilonacept are also demonstrated in clinical trials to be effective in certain diseases.^197^, ^198^, ^199^ One challenge of the utilization of these blockers revealed by the clinical trials is the off‐target effect, which lead to fatal infection. Li et al. come up with a strategy to overcome this limitation by using platelet microparticles for the accumulation of blockers in specific sites. This indicates a new direction to develop certain resort for the delivery of drugs to target particular organs and cells. Apart from inhibition of its activation, more researches for the precise functions of inflammasome in different conditions are needed for more purposeful and effective modification.

**TABLE 1:** Major IL‐1β antagonists and a part of relative clinical trials.

IL‐1β antagonists	Mechanism of actions	Condition	Phase of clinical trials	NCI number
Anakinra	IL‐1 receptor antagonist that binds to IL‐1 receptors with an affinity similar to that of IL‐1α and IL‐1β	Multiple myeloma	Phase 2	NCT03233776
		Covid19	Phase 3	NCT04680949
		Acute myocarditis	Phase 2	NCT03018834
			Phase 3	
		Type I diabetes	Phase 1	NCT00645840
			Phase 2	
		Acute myocardial infarction, heart failure	Phase 2	NCT01175018
		Gout, chronic kidney diseases	Phase 2	NCT02578394
			Phase 3	
		Metastatic colorectal, cancer	Phase 2	NCT02090101
		Acute gouty arthritis	Phase 2	NCT03002974
		Heart failure	Phase 1	NCT01300650
			Phase 2	
	Rheumatoid arthritis	Phase 3	NCT00117091
Canakinumab	Fully human anti‐IL‐1β monoclonal antibody that selectively blocks IL‐1β and has no cross‐reactivity with other characterized IL‐1 family members, including IL‐1α and IL‐1Ra	Non‐small cell lung cancer	Phase 2	NCT03968419
		Schnitzler syndrome	Phase 2	NCT01276522
		Type I diabetes	Phase 2	NCT00947427
		Diabetes mellitus, type 2	Phase 3	NCT04510493
		Cryopyrin‐associated periodic syndromes, familial cold autoinflammatory syndrome, Muckle‐Wells syndrome	Phase 3	NCT01576367
		Rheumatoid arthritis	Phase 2	NCT00554606
Rilonacept	A dimeric fusion protein consisting of the ligand‐binding domains of the extracellular portions of the human IL‐1R1 and IL‐1RAcP and neutralizes IL‐1	Recurrent pericarditis	Phase 3	NCT03737110
		Type 1 diabetes mellitus	Phase 1	NCT00962026
		Scleroderma, systemic sclerosis, diffuse scleroderma, diffuse systemic sclerosis	Phase 1, Phase 2	NCT01538719
		Familial Mediterranean fever	Phase 2	NCT00582907
		Muckle–Wells syndrome, Schnitzler syndrome	Phase 2	NCT01045772
		Gout	Phase 2	NCT00610363
		Recurrent pericarditis	Phase 2	NCT03980522
XOMA 052 (gevokizumab)	IL‐1β antibody that reduces the affinity of IL‐1β for its signaling receptor and coreceptor but not its decoy and soluble inhibitory receptors	Osteoarthritis	Phase 2	NCT01882491
		Diabetes mellitus type 1	Phase 2	NCT01788033
		Type 2 diabetes mellitus	Phase 2	NCT01144975

*Clinical trial data source*: clinicaltrials.gov.

In recent years, in addition to the traditional strategies to deal with inflammasome complex and cytokines, new approaches for controlling related diseases emerged with the advances in technologies. Engineered nanomaterials (ENMs) are widely used in biomedical field in recent years due to its interaction with body's immune system and capacity to activate immune responses. Many have become United States Food and Drug Administration (US FDA)‐approved or preclinically promising nanomedicines with their highly efficient delivery and increased safety.^200^, ^201^ ENMs can be applied to human in different ways including subcutaneous administration, inhalation administration, and systemic administration, leading to diametrically opposite outcomes, including immune activation, immune suppression or homeostasis turbulence, and it depends largely on antigen property and different physicochemical characteristics of ENMs.^202^ For the involvement and application of ENMs in inflammasome‐related diseases, evidence suggested that ENMs can be recognized by immune systems through PRR signaling, acting as PAMPs or DAMPs.^203^ And they may even have depot effect for the activation of inflammasomes.^204^ As for the precise mechanism of ENMs' participation in NLRP3 inflammasome activation, some studies suggested that it might be the generation of ROS^205^ and surface modification of ENMs^206^ that work. Scientists developed and applied an inflammation‐targeted Celastrol (Cel) nanodrug in both in vivo and in vitro experiments for the delivery of Cel, which has shown antirheumatic activity against RA. The result demonstrated this nanodrug can effectively inhibit the repolarization of macrophages toward M1 phenotype and alleviate the secretion of inflammatory cytokines.^207^

Over the past decades, efforts have also been made in the application of noncoding RNAs (ncRNAs) for disease treatment, several of which have already gained approval of US FDA. There are two major categories of ncRNA, microRNAs (miRNAs) and long ncRNAs (lncRNAs). The bio‐functions of these RNA sequences without the capacity of translating into protein remain largely unknown, but emerging evidence suggests that they can mediate RNA interference, thus regulate the function of immune system and the operation of inflammasomes. Therapeutic targeting of the naturally originated ncRNAs may promise great result for the treatment of inflammasome‐related diseases.

There lies two major advantages of targeting ncRNAs. First, according to central dogma of molecular biology, ncRNA therapeutics can target at several genes in one signal transduction pathway simultaneously. In the second place, as naturally occurring molecules rather than synthetic, ncRNAs have all the mechanisms in place for processing and downstream modifications.^208^ In the past few years, both miRNAs and lncRNAs were reported to involve in the activation of inflammasomes. For example, nuclear enriched abundant transcript 1 (*Neat1*), a lncRNA which is upregulated under hypoxic conditions in a HIF‐2α‐dependent manner, was identified to associate with NLRP3 inflammasome in mouse macrophage, and can also promote the activation of NLRC4 and AIM2 inflammasomes, enhance caspase‐1 activation and inflammatory cytokine production and pyroptotic cell death.^209^ The ortholog of *Neat1* in human, *NEAT1*, was proved to maintain the structural integrity of the paraspeckles.^210^ With regard to the application ncRNA in inflammasome‐related diseases, it was reported that miRNA circuits can orchestrate aberrant intestinal inflammasome of IBDs by targeting at NLRP3 inflammasome.^211^ In the mucosa of the gastrointestinal tract, the only monolayer ECs space out the gut microbiome from the body. To refrain from persistent inflammatory reactions and homeostasis imbalance of body's immune system, mammals have evolved a complex mucosal immune system with precise regulation of inflammasome and activation. IBDs, including Crohn's disease and ulcerative colitis, are hypothesized by be the result of a dysregulated innate immune response.^212^ Opinions toward the specific role of inflammasome in IBDs differed. In this study, scientists identified miR‐223 as not only a biomarker in patients with IBDs, but also a target for suppression of NLRP3 inflammasome activation.^211^

### 5.3 NLRP3 targeting strategies

Current treatments available for NLRP3‐related diseases target mainly at proinflammatory cytokines. Anakinra works as a human IL‐1 receptor antagonist, Canakinumab is a human anti‐IL‐1β monoclonal antibody developed by Novartis, and Rilonacept is a recently approved decoy IL‐1R for prevention and treatment of recurrent pericarditis.^213^, ^214^, ^215^ The defect is that these drugs only treat the symptoms but not the root cause. This highlights the importance of developing NLRP3‐specific therapeutics.

MCC950, also named CRID3, is a small‐molecule inhibitor for the NLRP3 inflammasome and is demonstrated to be effective in many NLRP3‐dependent inflammatory reactions. A study established mouse models of diseases related to NLRP3 activation or gain of function mutations of *nlrp3*, utilizing intraperitoneal injection of LPS and expression of the Muckle–Wells syndrome (MWS) associated mutation *Nlrp3^A350VneoR^* in myeloid lineage, respectively. MCC950 used as treatments showed effects in both in vivo and in vitro experiments. Follow‐up researches demonstrates the efficacy of MCC950 in many in vivo disease models.

Except the application value of MCC950 in disease models, what also interests us is the precise mechanism of MCC950's specific inhibition effect on NLRP3, the understanding of which can help to lower side effects of MCC950 and design more effective drugs. It has been confirmed that lysosomal destabilization, mitochondrial dysfunction and ROS production, tubulin acetylation and K^+^ efflux were proved to have nothing to do with the function mechanisms of MCC950. Based on the hypothesis that MCC950 target directly at NLRP3, the scientists applied a drug affinity responsive target stability assay and drew the conclusion that MCC950 protects NLRP3 in both active and inactive conformations, but cannot protect NEK7 from protease.^216^ Then they also used a synthesized photoaffinity probe to verify the conclusion that MCC950 interacts directly with NLRP3.^216^ To further decide the concrete domain that MCC950 bind with, full‐length NLRP3 was expressed in HEK293T as the vehicle. The result suggested that MCC950 can bind with the Walk B motif in NATCH domains, which is suggested to play a vital role in ATPase activity of NBD, thus blocking the hydrolysis capacity of NLRP3 on ATP for activation.^216^ A back‐to‐back study confirmed that MCC950 can close the ‘open’ conformation of activated NLRP3 in both wild type NLRP3 and autoinflammatory‐related mutants.^217^ Recently, relying on the utilization of cryo‐EM technology, scientists made a further step, elucidated the binding site of MCC950 on NLRP3 decamer and its ability to keep NLRP3 locked in an inactive conformation.^63^ These evidence support further researches to develop more NLRP3‐specific small inhibitory molecules. Besides MCC950, there are many other small molecules targeting NLRP3 inflammasome and are summarized in Table 2.

**TABLE 2:** NLRP3 inhibitors and related disease models.

NLRP3 inhibitors	Mechanisms of actions	Disease models	References
MCC950	Interact with the Walker B motif in the NACHT domain of NLRP3 and block NLRP3‐mediated ATP hydrolysis	PD, AD, asthma, IR lung injury, ARDS/COPD, atherosclerosis, hypertension, myocardial infarction, heart failure, colitis, acute liver failure, NASH, IR liver injury, liver fibrosis, insulin resistance, renal fibrosis, MWS, rheumatoid arthritis	^218^
CY‐09	Interact with the Walker A motif in the NACHT domain of NLRP3 and block NLRP3‐mediated ATP hydrolysis	AD, T2DM	^219^
Bay‐11‐7082	Block NF‐κB pathway and the priming of NLRP3 inflammasome	Psoriasis, diabetic neuropathy, cardiac ischemia–reperfusion injury, ATL	^220^, ^221^, ^222^, ^223^
Parthenolide	Block STAT3/NF‐κB pathway and the priming of NLRP3 inflammasome	Insulin resistance	^224^
BOT‐4‐one	The NLRP3 alkylation by BOT‐4‐one led to an impaired ATPase activity of NLRP3	Arthritis	^225^
OLT1177(Dapansutrile)	Reduce ATPase activity of t NLRP3, prevent NLRP3‐ASC and NLRP3‐caspase‐1 interaction	MS (EAE), colitis, allergic asthma, AD, gouty arthritis	^226^, ^227^, ^228^, ^229^, ^230^
INF39	Inhibit the interaction of NEK7‐NLRP3	IBD, T2DM, drug‐induced liver injury, acute colitis, pancreatitis	^231^
HS‐203873			
Tranilast	Directly bind to the NACHT domain of NLRP3 and suppresses the assembly of NLRP3 inflammasome	Atherosclerosis, COVID‐19, GDM, myocardial infarction	^232^, ^233^, ^234^, ^235^
Oridonin	Form a covalent bond with the cysteine 279 of NLRP3 in NACHT domain to block the interaction between NLRP3 and NEK7	Acute lung injury, pleurisy, myocardial infarction, liver fibrosis, acute liver injury, traumatic brain injury, noise‐induced hearing loss, reperfusion injury, silicosis	^236^, ^237^, ^238^, ^239^, ^240^, ^241^, ^242^, ^243^, ^244^
RRx‐001	Bind to cysteine 409 of NLRP3 via its bromoacetyl group and therefore blocks the interaction between NLRP3 and NEK7	LPS‐induced systemic inflammation, EAE, DSS‐induced colitis, MSU‐induced peritonitis	^245^
JC‐171	Interfere with NLRP3/ASC interaction	EAE	^246^
Rg3	Abrogate NEK7‐NLRP3 interaction	Myocardial infarction, ulcerative colitis, hepatic damage, myocardial hypertrophy, cisplatin‐induced kidney injury	^247^
C1‐27(&25)	Inhibit GSTO1‐1, which deglutathionylates cysteine 253 in (NEK7) to promote NLRP3 activation		
Artemisinin	Suppress interaction between NEK7 and NLRP3	AD, burn sepsis, tubulointerstitial inflammation, gout, IgA nephropathy, atherosclerosis, myocardial ischemia–reperfusion injury, dry eye disease, ulcerative colitis	^248^
KN3014	Target the interaction between NLRP3 and ASC through the PYD, reduce ASC‐speck formation		
β‐Carotene	Directly bind to the pyrin domain of NLRP3	Gouty arthritis	^249^
NIC‐0102 (27)	Induce the polyubiquitination of NLRP3, interfere with the NLRP3‐ASC interaction, and block ASC oligomerization	Ulcerative colitis	^250^
Wedelolactone	Promote the Ser/Thr phosphorylation of NLRP3 to inhibit inflammasome activation	Gouty arthritis, ulcerative colitis, cardiac fibrosis, collagen‐induced arthritis	^251^

Abbreviations: AD, Alzheimer's disease; AKI, acute kidney injury; ATL, adult T‐cell leukemia; DSS, dextran sodium sulphate; EAE, experimental autoimmune encephalomyelitis; GDM, gestational diabetes mellitus; IR, ionizing radiation; MS, multiple sclerosis; MWS, Muckle–Wells syndrome; NASH, nonalcoholic steatohepatitis; PD, Parkinson's disease; T2DM, type 2 diabetes mellitus.

In recent years, small molecules targeted at GSDMD also promise great results in preclinical disease models. GSDMD is the executor of pyroptosis and provide access for the release of proinflammatory cytokines. There is also evidence that activated GSDMD (NT‐GSDMD) can oligomerize and form pores in both cellular membrane and mitochondrial membrane, the latter result in dysfunction of mitochondria and production of mtROS.^252^ The important role of GSDMD in pyroptosis and more possibilities of its function mechanism make it an attractive target for therapeutics. Known inhibitors of GSDMD include disulfiram, necrosulfonamide, dimethyl fumarate, and so on.^253^ The defect of this strategy is that although blockade of GSDMD can inhibit the release of cytokines and cell membrane rupture, it cannot inhibit the maturation of IL‐1β and IL‐18.

As we have mentioned, blockade of NEK7 can lead to the downregulation of CASP1 activation and the release of IL‐1β, which indicates that we can use NEK7 as a therapeutic target for NLRP3 inflammasome‐related diseases. In fact, there already have been a few inhibitors targeting NEK7‐licensed NLRP3 inflammasome activation. Oridonin, which has been proved to block the NLRP3–NEK7 interfaces, can alleviate CASP1 activation and IL‐1β release caused by various NLRP3 agonist, such as LPS or ATP, as an important supplement to Oridonin's function mechanism on NF‐κB activation.^238^

Recent studies aimed at elucidating the mechanism of tumor immune escape found that ESCRT proteins play an important role in the repair of cell membrane wound, therefore protect tumor cell from T cell attack.^254^ Cytotoxic T lymphocytes and NK cells induce tumor cell death through FAS pathways and perforin. Cell death forms include necrosis, apoptosis, and pyroptosis. Earlier study has already demonstrated that granzyme A can cleave and activate GSDMB, a member of gasdermin protein family in a NLRP3‐dependent manner, then induce tumor cell pyroptosis and mediate tumor immunotherapy.^255^ It is reasonable to hypothesize that ESCRT can regulate cell fate affected by gasdermin pores, being supported by a study demonstrating that ESCRT is capable of repairing the membrane pores and reduce the release of IL‐1β after GSDMD activation.^118^ Together, ESCRT could allow effective targeting of NLRP3‐related inflammatory diseases.

Another strategy for prevention and alleviation of NLRP3‐related diseases is PTMs, which refer to any alteration in the amino acid sequence of the protein after its synthesis. PTMs mainly occur in the endoplasmic reticulum of the cell and continue in the Golgi bodies as well. As we have mentioned, before its priming, NLRP3 stays in an inactive state, partly due to various forms of PTMs including phosphorylation and ubiquitylation. For example, there are several conserved phosphorylation sites in NLRP3, and phosphorylation of LRR domain was proved to control activation of NLRP3.^256^ Emerging evidence supports the fact that PTMs are also an important mechanism in the regulation of NLRP3 activation. It is worth noting that as there exists multiple forms of PTMs, the modification role it plays in the activation of inflammasomes can be either enhancement or inhibition. We are looking forward to more comprehensive studies in this field. There are now a handful of drugs targeting the ubiquitin system being approved by the US FDA, and further testing of their effects on NLRP3‐related diseases might be significant for development of targeting therapy.

### 5.4 AIM2 targeting strategies

In recent years, research on AIM2 inflammasome has attracted great attention. One basic strategy to target the AIM2 inflammasome pathway is to block the interaction between dsDNA and AIM2 inflammasome. It was demonstrated that synthetic oligodeoxynucleotides (ODNs) containing TTAGGG motif can attenuate a variety of inflammatory responses and function through cytosolic nucleic acid‐sensing pathways.^257^ Scientists pretreated DCs and macrophages with ODN‐A151, an ssDNA species composed of four repeats of TTAGGG motif, and they observed abrogation of caspase‐1‐dependent IL‐1β and IL‐18 maturation after stimulation with dsDNA poly(dA:dT) and CMV. Next they lysed immortalized murine macrophages and incubated them with A151 without stimuli, revealing that A151 can interact with AIM2 competitively with dsDNA and achieve the goal of AIM2 activation inhibition.^257^

Although research focused on AIM2‐targeted therapies have been attractive in recent years, small molecules for AIM2‐specific inhibition have not been reported. Yet there are still some chemical compounds demonstrated to have inhibitory effects on AIM2, most of which are from natural origins, providing evidence for development of AIM2‐specific inhibitory drugs. Radiation therapy are commonly used in the treatment for patients suffering from cancer. Due to the damage of radiation to variety of cells and organs, among which lung and gastrointestinal tract are the most sensitive, applications of radiation therapy are greatly limited. Radiation‐induced lung injury (RILI) is a severe complication of radiotherapy for thoracic tumors. The progression of RILI can be divided into the acute radiation pneumonitis and radiation‐induced lung fibrosis in the late stage, both being related to the onset of inflammatory responses. Emerging evidence suggests that AIM2 inflammasome‐induced pyroptosis in ECs and macrophages plays a role in the inflammatory responses in RILI.^258^ Andrographolide is an active component with antibacterial, anti‐inflammatory and anticancer effects and was demonstrated to contributes to amelioration of RILI. Mice were first exposed to 18 Gy radiation and then treated with Andrographolide. Results demonstrated that Andrographolide can alleviate inflammatory lung damage caused by radiation, and can therefore be identified as a novel potential protective agent for RILI.^259^ Emerging evidence also supports the critical role of AIM2 inflammasome in ischemic brain damage, for instance, ischemic stroke.^260^ Histone deacetylases 3 (HDAC3) has antitumor efficacy in various cancers through histone modification of p53 and Bax and is reported to mediate inflammatory response and LPS tolerance in human macrophages and monocytes.^261^ RGFP966, a selective HDAC3 inhibitor, can cross the blood–brain barrier.^262^ Scientists generated AIM2 knockout mice and administrated RGFP966 intraperitoneally at 10 mg/kg after ischemic stroke injury. It was then demonstrated that RGFP966 can on the one hand decrease inflammasome expression in both in vivo and in vitro experiments, and on the other hand have protective effects on ischemic stroke. Further experiment suggested that RGFP966 treatment does not have protective effects on AIM2^−/−^ mice, together we can draw the conclusion that RGFP966 can alleviate ischemic brain injury through regulation of AIM2 expression.^263^ Besides, fructose‐arginine, a nonsaponin molecule of Korean Red Ginseng extract has also been reported to have positive effects in diseases associated with AIM2 activation for its being able to attenuate AIM2 activation in both animal models and cultured cells.^264^

## 6 CONCLUSION AND FUTURE PERSPECTIVES

In this review, we first give a brief introduction of the activation process of NLPR3, AIM2, and NLRC4 inflammasomes. Growing evidence supports the inference that inflammasomes can be activated by a broad spectrum of stimuli through several different pathways. Contrary to previous thought that inflammasomes rely only on caspase‐1 in caspase family proteins and will inevitably lead to cell pyroptosis, it has been demonstrated that caspase‐4/5/11 characterize noncanonical inflammasome activation pathway^30^, ^31^ and that TLR4–TRIF–RIPK1–FADD–CASP8 signaling pathway also contributes to the collective response of inflammasomes to danger signals.^36^ Diverse cell fates as the result of inflammasome activation also indicate the necessity of precise regulation of inflammasome in the treatment of related diseases.

For the structure analysis of inflammasomes, advances emerged in recent years toward two major challenges in this field, purification of proteins and technologies to analyze three‐dimensional structure of them. Details of the inflammasome structure in both inactive and active states extend our understanding of the specific functions of different domains and motifs. For NLRP3, especially, cryo‐EM revealed the double‐ring cages structure of inactive mouse NLRP3, the decamer‐oligomer structure of human full‐length NLRP3 and the 10/11 fold disk structure with coexpression of NEK7 and NLRP3 jointly provide us with insight into the accurate binding cite and working mechanism of MCC950, the NLRP3‐specific inhibitor.^62^, ^63^, ^64^

Inflammasome as an important part of innate immunity, is proved to act as a bridge between innate and adaptive immunity for indirect function through proinflammatory cytokines and direct function to regulate the development of lymphocytes.^101^ The basic function of inflammasome can be briefly summarized as IL‐1β and IL‐18 release and cell pyroptosis, while other distinct cell fates are also involved in the protection and injury mechanisms. In recent years, the status of inflammasome in TAI attracted great attention, as it has been proved to play a dual role in the pathogenesis of cancers.^120^ In fact, combination of CAR‐T therapy and regulation of inflammasome in cancer treatment are proved to ensure and strengthen antitumor effects of the treatment.^265^

Therapeutic targets of inflammasome‐related diseases evolved with extended understanding of inflammasome activation, structure and biological functions. In this review, we give a retrospection of proinflammatory cytokine targeted therapeutics and the advances in MCC950 application and the development of other small molecules and chemical compounds that promised great results in disease models. We also highlight the prospect to target other proteins in inflammasome pathway including gasdermin family proteins and the ESCRT system for membrane repairment. In addition, application of nanomaterial and ncRNA may possess a certain value.

## AUTHOR CONTRIBUTIONS

Yali Dai, Jing Zhou, and Chunmeng Shi contributed to the discussion of the outline and content of this review and edited the manuscript before submission. Yali Dai drafted the manuscript and illustrated the figures. All authors have read the manuscript before submission and approved the final version of this manuscript.

## CONFLICT OF INTEREST STATEMENT

The authors declare that they have no conflict of interest.

## ETHICS STATEMENT

No ethical approval was required for this study.

## Data Availability

Not applicable.
